# Prevalence of malnutrition and risk of undernutrition in hospitalised children with liver disease

**DOI:** 10.1017/jns.2017.56

**Published:** 2017-10-30

**Authors:** Ronghua Yu, Yizhong Wang, Yongmei Xiao, Lili Mo, Aishu Liu, Dan Li, Ting Ge, Guangjun Yu, Ting Zhang

**Affiliations:** 1 Department of Gastroenterology, Hepatology, and Nutrition, Shanghai Children's Hospital, Shanghai Jiao Tong University, Shanghai 200062, People's Republic of China; 2 Department of Children's Healthcare, Shanghai Children's Hospital, Shanghai Jiao Tong University, Shanghai 200062, People's Republic of China

**Keywords:** Liver disease, Children, Malnutrition, Undernutrition, ALT, alanine transaminase, HAZ, height-for-age *Z*-score, WAZ, weight-for-age *Z*-score, WHZ, weight-for-height *Z*-score

## Abstract

Nutritional status of 380 hospitalised children aged from 1 month to 5 years with liver disease was evaluated in a single paediatric centre. The total prevalence of stunting (height-for-age *Z* (HAZ) < −2), underweight (weight-for-age *Z* (WAZ) < −2) and wasting (weight-for-height *Z* < −2) was 9·8, 9·0 and 7·9 %, respectively. The overall nutritional risk (−2 ≤ *Z* < −1) of stunting, underweight and wasting was 11·8, 12·9 and 12·6 %. The prevalence of undernutrition was significantly higher in children with cholestasis than children without cholestasis (stunting, 17·5 %/4·4 %, *P* < 0·001, and underweight, 14·9 %/4·9 %, *P* < 0·001). HAZ and WAZ scores were significantly higher in children without cholestasis than children with cholestasis (0·58 (sd 1·59)/−0·68 (sd 1·99), *P* < 0·001, and 0·37 (sd 1·35)/−0·47 (sd 1·75), *P* < 0·001). Further multivariate logistic regression analysis strengthened the evidence that cholestasis was significantly associated with undernutrition of stunting (OR = 4·18, *P* = 0·002) and underweight (OR = 3·26, *P* = 0·008), and suggested that the prevalence of stunting caused by infection was lower than other aetiologies in hospitalised children with liver disease (OR = 0·10, *P* = 0·002). We concluded that a high prevalence of malnutrition and risk of undernutrition presents in hospitalised young children with liver disease, especially in children with cholestasis. Nutrition assessment is recommended for hospitalised children with liver disease.

Malnutrition is a state of nutrition in which a deficiency or excess, or imbalance of energy, protein and other nutrients causes measurable adverse effects on tissue/body form, body function and clinical outcome^(^^1^^)^. The human liver is an important regulator of metabolism, storage, synthesis and absorption of nutrients^(^^2^^)^. Nutritional deficiencies are common among people with chronic liver disease, especially in children^(^^3^^–^^5^^)^. The malnutrition prevalence in children with chronic liver disease may range from 9·1 to 71·1 % depending on the severity of liver disease^(^^5^^–^^7^^)^. The development of malnutrition in childhood liver disease is complex and involves multiple mechanisms, including decreased dietary intake, impaired nutrient digestion and absorption, increased energy requirements and disordered substrate use^(^^8^^–^^10^^)^. Liver disease patients with malnutrition have greater incidence of complications^(^^11^^,^^12^^)^. They have higher risk of infections, longer hospital stay and cost, and increased mortality after liver transplantation compared with well-nourished patients^(^^13^^–^^16^^)^. In addition, patients’ nutrition status is one of the effective factors on the prognostic variables during liver transplantation^(^^16^^,^^17^^)^. Therefore, it is very important to assess the nutrition status of paediatric patients with chronic disease, including chronic liver disease.

Currently, there are limited studies focused on the nutrition status of children with liver disease in China. The present study assessed the nutritional status, and evaluated the prevalence of malnutrition and risk of undernutrition of hospitalised children with liver disease at the Shanghai Children's Hospital.

## Methods

### Study population

This single-centre, cross-sectional prospective study was performed at the Department of Gastroenterology, Hepatology, and Nutrition, the Shanghai Children's Hospital, China. Patients were recruited from December 2013 to March 2016. Hospitalised patients, aged from 1 month to 5 years, with a diagnosis of liver disease were recruited in the study period. The diagnosis of liver disease is defined as satisfying at least one of the following clinical manifestations: hepatomegaly, jaundice and alanine transaminase (ALT) >80 U/l. This study was conducted according to the guidelines laid down in the Declaration of Helsinki and all procedures involving human subjects were approved by the Shanghai Children's Hospital Institutional Review Board. Written informed consent was obtained from all the guardians of participants.

### Data collection

The data from patients’ electronic medical records were collected, including demographics (age, sex) and clinical data (laboratory values and imaging study results). According to the recommendations of the North American Society for Pediatric Gastroenterology, Hepatology and Nutrition^(^^18^^)^, we defined an abnormal direct bilirubin as a value greater than 17·1 µmol/l if the total bilirubin is less than 85·5 µmol/l, or a value of direct bilirubin that represents more than 20 % of the total bilirubin if the total bilirubin is greater than 85·5 µmol/l. In the present study, the patients were divided into the cholestasis group and the non-cholestasis group according to the direct bilirubin value.

Anthropometric measures were completed within 24 h of admission, including weight and length/height. Children younger than 3 years and weighing up to 10 kg were measured in an electronic baby scale (SECA 335) without clothes and nappy; an electronic personal scale (SECA 704s) was used to measure children older than 3 years. Patients were evaluated barefoot and, usually, only with underwear. The equipment was checked before use. Data were analysed using the software Epi info version 3.5.4 (Centers for Disease Control and Prevention), and converted to the following variables: height-for-age *Z*-score (HAZ), weight-for-age *Z*-score (WAZ) and weight-for-height *Z*-score (WHZ). Nutrition risk and malnutrition were defined according to *Z*-score (nutrition risk: −2 ≤ *Z* < −1; moderate malnutrition: −3 ≤ *Z* < −2; severe malnutrition: *Z* < −3), while stunting, underweight and wasting were defined according to HAZ (HAZ < −2), WAZ (WAZ < −2) and WHZ (WHZ < −2), respectively.

### Statistical analysis

Statistical analysis was performed by SPSS 20.0 software. The data are shown as medians and interquartile ranges (25th–75th percentile), or means with standard deviations, and compared using the non-parametric Mann–Whitney test. The distribution of dichotomous variables was compared using the *χ*^2^ test. The logistic regression model was generated using variables including sex, age, ALT > 80 U/l, cholestasis and aetiology, in which aetiology was a categorical variable. The method of logistic regression model was ‘enter’. A *P* value of <0·05 was considered to be statistically significant.

## Results

### Clinical characteristics

A total of 380 hospitalised children with liver disease were evaluated, and their main clinical characteristics are shown in Table 1. The median age was 5 months (interquartile range 2–20 months), and 59·5 % (226/380) of them were male. As for the age distribution, infants, toddlers and preschool children accounted for 67·1 % (255/380), 19·2 % (73/380) and 13·7 % (52/380), respectively. In 380 children, 154 (40·5 %) subjects had cholestasis, 213 (56·1 %) had hepatomegaly, 191 (50·3 %) subjects had jaundice, and 262 (68·9 %) cases had ALT more than 80 U/l. The aetiologies of liver disease were: biliary atresia in twenty-four patients (6·3 %), Alagille syndrome in six (1·6 %), progressive familial intrahepatic cholestasis in eight (2·1 %), glycogen storage disease in eight (2·1 %), Wilson disease in five (1·3 %), neonatal intrahepatic cholestasis in seven (1·8 %), viruses and other infectious hepatitis in 121 (31·8 %), and other diagnoses in thirty-four (8·9 %). The aetiologies of 167 (43·9 %) cases were undefined according to the medical record.

**Table 1.** Clinical characteristics of 380 hospitalised children with liver disease (Mean values and interquartile ranges (IQR); numbers of children and percentages)

Characteristics	Mean	IQR	*n*	%
Sex				
Male			266	59·5
Female			154	40·5
Age (months)	5	2, 20		
Hepatomegaly			213	56·1
Cholestasis			154	40·5
ALT (U/l)	133	64, 255		
Total bilirubin (μmol/l)	18·52	7·90, 129·43		
Direct bilirubin (μmol/l)	6·00	1·90, 65·10		
Aetiology				
Structural abnormalities			32	8·4
Biliary atresia			26	6·8
Others			6	1·6
Genetic/metabolic disorders			49	12·9
Alagille syndrome			7	1·8
Progressive familial intrahepatic cholestasis			8	2·1
Glycogen storage disease			8	2·1
Wilson disease			6	1·6
Neonatal intrahepatic cholestasis			8	2·1
Others			12	3·2
Infectious diseases			151	39·7
Cytomegalovirus			54	14·2
Epstein–Barr virus			51	13·4
Hepatitis B virus			7	1·8
Others			46	12·1
Others			32	8·4
Unknown			125	32·9

ALT, alanine transaminase.

### Prevalence of nutritional risk and malnutrition

As shown in Table 2, the nutritional risk (−2 ≤ *Z* < −1) of stunting, underweight and wasting was 11·8 % (45/380), 12·9 % (49/380) and 12·6 % (48/380), respectively. The total malnutrition prevalence of stunting (HAZ < −2), underweight (WAZ < −2) and wasting (WHZ < −2) was 9·8 % (37/380), 9·0 % (34/380) and 7·9 % (30/380). Among the malnutrition patients, moderate malnutrition (−3 ≤ *Z* < −2) of stunting, underweight and wasting was observed in seventeen (4·5 %, 17/380), twenty (5·3 %, 20/380) and sixteen (4·2 %, 16/380) patients, while severe malnutrition (*Z* < −3) was found in twenty (5·3 %, 20/380), fourteen (3·7 %, 14/380) and fourteen (3·7 %, 14/380) subjects, respectively.

**Table 2.** Prevalence of nutritional risk and malnutrition of the patients with liver disease (Numbers of children and percentages)

	Stunting		Underweight		Wasting	
Nutrition status	*n*	%	*n*	%	*n*	%
Nutritional risk (−2 ≤ *Z* < −1)	45	11·8	49	12·9	48	12·6
Moderate malnutrition (−3 ≤ *Z* < −2)	17	4·5	20	5·3	16	4·2
Severe malnutrition (*Z* < −3)	20	5·3	14	3·7	14	3·7
Total	82	21·6	83	21·9	78	20·5

### Factors associated with nutritional risk and malnutrition

The 380 patients were divided into the cholestasis group and the non-cholestasis group according to the definition of cholestasis. The prevalence of stunting, underweight and wasting in children with cholestasis was 17·5 % (27/154), 14·9 % (23/154) and 7·9 % (13/152), while the prevalence of stunting, underweight and wasting in children without cholestasis was 4·4 % (10/226), 4·9 % (11/226) and 7·6 % (17/223), respectively (Table 3). The differences in stunting and underweight between the cholestasis group and non-cholestasis group were statistically significant (*P* < 0·001, *P* = 0·001); however, there was no statistically significant difference between the two groups in terms of wasting (*P* = 0·745; Table 3). The HAZ, WAZ and WHZ scores of the whole study patients were 0·07 (sd 1·86), 0·03 (sd 1·58) and −0·08 (sd 1·57), respectively (Table 4). The *Z*-scores of HAZ, WAZ and WHZ of the cholestasis group were lower than the non-cholestasis group, and the differences in HAZ (0·58 (sd 1·59)/−0·68 (sd 1·99), *P* < 0·001) and WAZ (0·37 (sd 1·35)/−0·47 (sd 1·75), *P* < 0·001) were statistically significant, but there was no significant difference between the two group in terms of WHZ (−0·05 (sd 1·50)/−0·11 (sd 1·66), *P* = 0·722; Table 4). Further multivariate logistic regression analysis strengthened the evidence that cholestasis was significantly associated with undernutrition of stunting (OR = 4·18, *P* = 0·002) and underweight (OR = 3·26, *P* = 0·008), and suggested that the prevalence of stunting caused by infection was lower than other aetiologies in hospitalised children with liver disease (OR = 0·10, *P* = 0·002) (Table 5).

**Table 3.** Comparison of malnutrition prevalence in patients with and without cholestasis (Numbers of children and percentages)

	Stunting		Underweight		Wasting	
	*n*/*N*	%	*n*/*N*	%	*n*/*N*	%
With cholestasis	27/154	17·5	23/154	14·9	13/152	8·6
Without cholestasis	10/226	4·4	11/226	4·9	17/223	7·6
*χ*^2^	17·91		11·40		0·11	
P*	<0·001		0·001		0·745	

* χ^2^ Test.

**Table 4.** *Z*-scores of the anthropometric results of the whole group of patients and stratified according to the presence of cholestasis (*Z*-scores and standard deviations)

	HAZ		WAZ		WHZ	
	*Z*	sd	*Z*	sd	*Z*	sd
General (380 children)	0·07	1·86	0·03	1·58	−0·08	1·57
With cholestasis (154 children)	−0·68	1·99	−0·47	1·75	−0·11*	1·66
Without cholestasis (226 children)	0·58	1·59	0·37	1·35	−0·05^†^	1·50
*P*‡	<0·001		<0·001		0·722	

HAZ, height-for-age *Z*-score; WAZ, weight-for-age *Z*-score; WHZ, weight-for-height *Z*-score.

* 152 Children.

† 223 Children.

‡ Independent-samples *t* test (cholestasis group and non-cholestasis group).

**Table 5.** Multivariate logistic regression analysis of variables associated with undernutrition (Odds ratios and 95 % confidence intervals)

Variable	Stunting			Underweight			Wasting		
	OR	95 % CI	*P*	OR	95 % CI	*P*	OR	95 % CI	*P*
Male (yes/no)	0·95	0·46, 1·98	0·899	1·34	0·62, 2·88	0·459	0·86	0·40, 1·86	0·701
Age (continuous)	1·02	0·99, 1·04	0·211	1·00	0·98, 1·03	0·812	1·01	0·99, 1·03	0·338
ALT > 80 U/l (yes/no)	0·69	0·32, 1·46	0·327	0·96	0·43, 2·12	0·915	1·77	0·35, 1·86	0·505
Cholestasis (yes/no)	4·18	1·72, 10·15	0·002	3·26	1·36, 7·79	0·008	1·32	0·52, 3·32	0·556
Aetiology*									
Others (reference group)	1		0·006	1		0·029	1		0·248
Structural abnormalities	0·37	0·10, 1·39	0·141	0·00	0·00	0·998	2·54	0·74, 8·72	0·140
Genetic/metabolic disorders	1·21	0·50, 2·95	0·672	2·04	0·84, 4·95	0·116	0·57	0·11, 2·84	0·491
Infectious diseases	0·10	0·02, 0·44	0·002	0·35	0·12, 1·01	0·052	1·58	0·63, 3·98	0·331

ALT, alanine transaminase.

* Aetiology: 1, structural abnormalities; 2, genetic/metabolic disorders; 3, infectious diseases; 4, others.

## Discussion

Nutritional deficiencies are common in children with chronic liver disease, especially when cholestasis is present and its onset occurs in the first year of life. Therefore, nutritional evaluation of these children is essential. In the present study, in order to provide reference for rational application of nutritional support, we examined the nutritional status of hospitalised children under 5 years old with a diagnosis of liver disease in a single paediatric centre in China. Finally, 380 in-patients were recruited in the study period. Clinical characteristics showed that 40·5 % subjects had cholestasis, 56·1 % cases had hepatomegaly, 50·3 % subjects had jaundice, and 262 (68·9 %) cases had ALT more than 80 U/l. The main aetiologies of liver disease were structural abnormalities, genetic/metabolic disorders and infectious disease. Nutrition assessment results showed that the prevalence of stunting, underweight and wasting was 9·8, 9·0% and 7·9 %, respectively, which was greater than the overall malnutrition rate of paediatric in-patients in Shanghai (stunting 7·1 %, underweight 5·5 % and wasting 5·2 %, respectively) reported in 2007^(^^19^^)^, and much greater than the overall rate of malnutrition in children under 5 years of age in cities of China in 2002 (stunting 4·9 %, underweight 3·1 % and wasting 1·8 %, respectively)^(^^20^^)^. Severe malnutrition in wasting was greater than that in paediatric hospitalised patients in Germany (3·7 *v.* 1·7 %)^(^^21^^)^. It has been suggested that liver disease is more likely to cause malnutrition, especially severe malnutrition, than other diseases in children. However, the malnutrition prevalence was lower than in a recent study in Brazil (stunting 50·0 %, underweight 27·3 % and wasting 11·1 %, respectively)^(^^5^^)^. The difference may be attributed to the course and severity of the liver disease.

To further investigate the factors associated with malnutrition and risk of undernutrition in children with liver disease, 380 patients were divided into a cholestasis group and a non-cholestasis group. Although there was no significant difference between the cholestasis group and non-cholestasis group in terms of wasting, the prevalence of stunting and underweight in the cholestasis group was significantly higher than in the non-cholestasis group. However, the prevalence of stunting (17·5 %/4·4 %) and underweight (14·9 %/4·9 %) in the two groups was lower than that observed in an earlier study (64·3 %/18·2 %, 57·2 %/9·1 %)^(^^22^^)^. Similarly, the prevalence of stunting (17·5 %), underweight (14·9 %) and wasting (8·6 %) in the cholestasis group was lower than in another similar study in Brazil (stunting 30·8 %, underweight 33·0 % and wasting 12·1 %, respectively)^(^^5^^)^. The reason may be that we have different criteria for the inclusion of cholestasis. In addition to cholestasis, multivariate logistic regression analysis suggested that earlier age and hepatomegaly were independent factors that were significantly associated with nutritional risk of underweight in hospitalised children with liver disease.

The authors are aware of limitations of the present study. In this study, children with liver disease had a variety of causes, and in different course and severity of disease, while the nutritional assessment was performed in a single moment. Another limitation is that body weight can be overestimated in cases of visceromegaly, ascites and peripheral oedema, at least in part, so weight for age, weight for height and BMI may underestimate the degree of undernutrition in children with chronic liver disease. The European Society of Clinical Nutrition and Metabolism (ESPEN) 2006 guideline recommends using Subjective Global Assessment and anthropometry to identify patients with cirrhosis who are at risk of undernutrition^(^^23^^)^. Some studies suggest that triceps skinfold thickness, mid-arm circumference, arm muscle circumference, global assessment of nutritional status and handgrip strength are indices which can better reflect the nutritional risk and the severity of cirrhosis in patients with chronic liver disease^(^^5^^,^^24^^,^^25^^)^. Thus, it is necessary to include more sensitive parameters to better evaluate the nutritional status of children with liver disease in the future.

In conclusion, the malnutrition prevalence in hospitalised children with liver disease is high, especially in children with cholestasis. In order to shorten the hospitalisation time and improve the clinical outcome, nutrition assessment and timely nutritional support are recommended for hospitalised children with liver disease.
